# Seroprevalence of anti-SARS-CoV-2 antibodies among staff at primary healthcare institutions in Prishtina

**DOI:** 10.1186/s12879-022-07038-6

**Published:** 2022-01-16

**Authors:** Rrezart Halili, Jeta Bunjaku, Bujar Gashi, Teuta Hoxha, Agron Kamberi, Nexhmedin Hoti, Riaz Agahi, Vlora Basha, Visar Berisha, Ilir Hoxha

**Affiliations:** 1 Main Family Medical Centre, Fehmi Agani Rd, Prishtina, 10000 Kosovo; 2 Evidence Synthesis Group, Prishtina Starts, Veternik, Prishtina, 10000 Kosovo; 3 Research Department, Heimerer College, Veranda D4, Hyrja C dhe D, Lagja Kalabri, Prishtina, 10000 Kosovo; 4 Index Kosova, 32A Gazmend Zajmi, Prishtina, 10000 Kosovo; 5 The Dartmouth Institute for Health Policy and Clinical Practice, Geisel School of Medicine at Dartmouth, 1 Medical Center Drive, Lebanon, NH 03766 USA

**Keywords:** COVID-19, Epidemiology, Healthcare workers, Infectious diseases, Public Health, Serology

## Abstract

**Background:**

Many studies examined the spread of SARS-CoV-2 within populations using seroprevalence. Healthcare workers are a high-risk population due to patient contact, and studies are needed to examine seroprevalence of SARS-CoV-2 antibodies among healthcare workers. Our study investigates the seroprevalence of anti-SARS-CoV-2 antibodies among staff at primary healthcare institutions in Prishtina, and factors associated with seroprevalence.

**Methods:**

We carried out a cross-sectional survey including SARS-CoV-2 serological testing and questionnaires with primary healthcare workers from primary healthcare facilities in the Prishtina, the capital city of Kosovo. We calculated prevalence of anti-SARS-CoV-2 antibodies, and of self-reported positive PCR test among primary healthcare workers, as well as crude and adjusted ORs for explanatory factors.

**Results:**

Eighty-three of the healthcare workers (17.47%) tested positive for SARS-CoV-2 antibodies IgG or IgM, while 231 (48.63%) either had antibodies or a previous positive PCR test. Odds of seropositivity were affected by male gender (OR 2.08, 95% CI 1.20, 3.61), and infected family members (OR 3.61, 95% CI 2.25, 5.79) of healthcare workers. Higher education, being part of larger families and having infected family members gave higher odds of positive PCR test and seropositivity. Other healthcare workers had lower odds of positive PCR test and seropositivity than physicians.

**Conclusion:**

Over 17% of healthcare workers were seropositive for SARS-CoV-2 antibodies and close to half of them were either seropositive or PCR self-reported positive test. Several factors are associated with decreased and increased odds for such outcomes. These findings should be explored further and addressed to Kosovo policy makers, and assist them to intensify vaccination efforts, and maintain control measures until we achieve herd immunity.

## Key messages

- Primary healthcare workers have a prevalence of 17.47% for SARS-CoV-2 antibodies
- Almost half of those surveyed had either tested positive by PCR or had antibodies
- We did not find a significant association with adherence to protective measures
- Infected family members significantly increased the odds of seropositivity

## Introduction

COVID-19 is a novel viral disease caused by SARS-CoV-2 originating from Wuhan, China in December 2019 [1]. This infectious disease was named COVID-19 by the World Health Organization at the beginning of last year. At the time of writing, more than 315 million cases and more than 5.5 million deaths around the world have been confirmed [2]. SARS-CoV-2 rapidly spread worldwide, which continues to pose a major challenge and is an ongoing threat for public health and healthcare systems. In several countries, the demand for medical care exceeds the available resources, requiring stakeholders to reorganize the medical landscape [3].

During pandemics, everyone can potentially be infected, but healthcare workers (HCWs) are at a particularly high risk of infection, due to direct and indirect contact with patients [4]. As SARS-CoV-2 is transmitted via airborne droplet infection and indirect contact with COVID-19 patients, all kinds of HCWs are at high risk of infection [5]. Healthcare staff face numerous challenges, including: increased workload created by outbreaks, fear of contagion for themselves and their families, working with new and frequently changing protocols and personal protective equipment (PPE), caring for patients who are very sick and quickly deteriorating, and caring for colleagues who become ill [6]. Many studies have examined seroprevalence among HCWs. For example, some studies from different countries have reported low seroprevalence of COVID-19 in HCWs who work in pediatric hospitals [7, 8], tertiary hospitals [9, 10], university or academic hospitals [11, 12] and emergency HCWs [13]. Some studies show that measurement of seroprevalence in SARS-CoV-2 in frontline HCWs can give highly varied results based on the country or region of testing, healthcare role and when testing was carried out [14–16].

Kosovo was among the last countries in the region and Europe to be hit by the coronavirus pandemic, with the first cases confirmed on 13th March 2020 [17]. Prishtina, as a capital city and a more populated area, has recorded more cases of the virus than other cities [18]. As a result, the frontline health workforce has experienced a high workload during the pandemic, along with multiple psychosocial stressors, which may affect their mental and emotional health, leading to burnout symptoms [19].

SARS-CoV-2 infection is followed by an antibody response with IgG and IgM antibodies, and therefore serological tests can provide more information on SARS-CoV-2. Antibody tests are potentially useful for detecting previous infection when measured 15 or more days after the onset of symptoms, but the duration of elevated antibody levels is currently unknown [20].

In Kosovo, primary healthcare facilities are the first contact for most patients. Given their continuous exposure to the virus, extensive and continuous testing of primary HCWs is a necessity. Kosovo has not been an exception to this requirement, as the pandemic has posed considerable danger to HCWs, particularly primary HCWs. This is the first study to date examining seroprevalence in primary HCWs in Kosovo. Studies elsewhere in the world have reported 2.6% [21] and 9.17% [22] seropositivity of COVID-19 antibodies among primary HCWs. This study aims to determine the prevalence of positive anti-SARS-CoV-2 antibodies among primary HCWs in the municipality of Prishtina, and its association with different demographic, epidemiological factors and health behaviors.

## Methods

### Study design and setting

We used cross-sectional survey data, collected from municipal workers (healthcare workers and administration), to determine the seroprevalence of anti-SARS-CoV-2 antibodies among primary healthcare workers in Prishtina. The data was collected between 14 October to 17 of December 2020. This survey collected information on demographics, socioeconomic status, educational background, exposure to the COVID-19 virus in the workplace, protective measures applied, COVID-19 symptoms experienced, and their health status (before, during, and after COVID-19 virus disease). The questionnaire for the survey was tested and revised before the data collection. All interviewers, prior to data collection had received training in study methodology and questionnaire implementation, as well as confidentiality and ethical behavior. The interviewers provided respondents with a phone number, and if the respondent was unable to answer the questions at the time of testing, the interviewer would schedule another convenient appointment. The interview data were filled in the questionnaire by hand (paper and pencil).

### Sampling, participants, testing and data collection

**Fig. 1:**
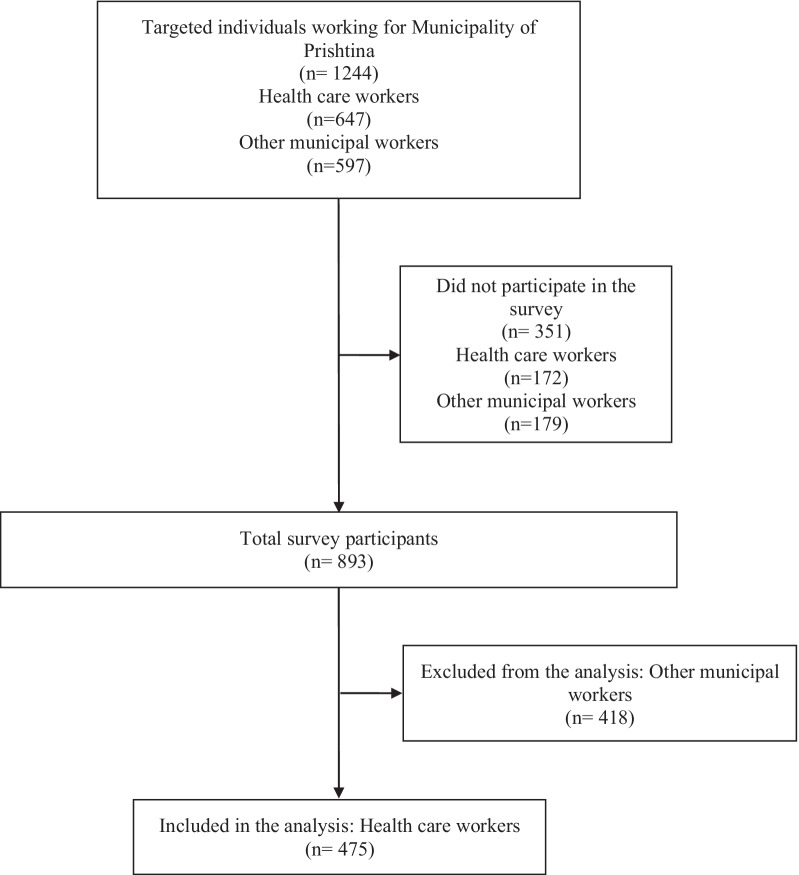
Sample selection flow chart

The sample was consecutive, i.e. survey targeted all healthcare and administration workers of the Municipality of Prishtina, who were invited to be part of the study. Contact data was provided by the Municipality of Prishtina. The sample selected for this study included only HCWs. A flow chart for the sample selection process is presented in Fig. 1. One hundred and seventy-two HCWs did not participate because they refused, were on leave, were busy, or it was not possible to contact them due to no connection or an incorrect phone number. Empirical data were collected from 475 HCWs from 32 primary healthcare facilities.

Study participants were invited to be tested for anti-SARS-CoV-2 antibodies. Two immunoassay tests were done to detect IgM and IgG antibodies. The testing was performed using VIDAS® SARS-CoV-2 by bioMérieux. Blood samples were drawn within the healthcare facility for all participants of the study. Post-test, information was collected through a telephone interview that allowed interpersonal communication without the need for a face-to-face meeting with the respondent. The respondents were asked if they were willing to respond to some questions related to their health condition and exposure to the COVID-19 virus. All individuals who agreed to participate were asked for their informed consent. Informed consent was obtained verbally over the telephone before starting the interview. They were informed that their participation is voluntary, and each participant was given the option to refuse to participate, to answer any question, or to terminate the interview and participation at any time. Ethical approval for the study was received from ethics committee of the Kosovo Doctors Chamber (12-08-2020, NR. REF. 8/2020). The study was carried out in accordance with the guidelines of the Helsinki Declaration for human participant data.

### Outcome measures

The primary pre-specified outcomes were COVID-19 prevalence measures, including either positive IgG or IgM SARS-CoV-2 antibodies test, or/and self-reported PCR positive test. A serological (IgM or IgG) test was considered positive in case of “i” (index value result with VIDAS® SARS-CoV-2) was equal or larger than 1. A self-reported PCR test was considered positive in case the respondent reported that they had undergone a positive PCR test. From both of these outcomes we derived a third outcome variable that would register that a respondent had a serological or PCR self-reported positive test in order to register prevalence for COVID-19 according to any of the sources of data available. The most reliable outcome measure is serologic prevalence as we have had access directly to test results. We use the other two outcome measures to complete the picture on prevalence of COVID-19 among HCWs, knowing serological testing can turn negative at early stages of disease or after some time has passed.

Additional outcome measures were the crude and adjusted OR of COVID-19 seroprevalence or/and self-reported PCR positive test result with respect to different characteristics such as level of education, gender, residence, protective behaviors towards COVID-19, type of healthcare worker, etc.

### Statistical analysis

We first calculated the prevalence of SARS COVID-19 using IgG, IgM, or/and self-reported PCR test result measures (Table 1). Then we performed a descriptive analysis of SARS-CoV-2 prevalence against several categories of variables. Crude univariable logistic regression was performed to test the unadjusted associations of variables with odds for seroprevalence. Then all the variables with a p-value < 0.10 representing differences that could potentially influence the seroprevalence were included in multiple logistic regression. We tested for collinearity in the adjusted models. Such tests showed no indication of collinearity in examination of Variance Inflation Factor (VIF) for each of variables included in the models.

**Table 1:** Prevalence of COVID-19 in healthcare workers

Summary results of prevalence of COVID-19 among healthcare workers				
	Events	%	Mean	SD
IgM or IgG positive	83	17.47		
IgM positive	53	11.16		
IgM value			0.6312358	2.120718
IgG positive	73	15.37		
IgG value			1.718425	5.384138
PCR positive	189	39.79		
PCR or IgM or IgG positive	231	48.63		
Total	475			

Analyses were performed using STATA, release V.15 IC (StataCorp).

## Results

### Study sample

All study participants included were HCWs working in primary care sector in municipality of Prishtina, namely doctors (30.7%), nurses (48.4%), and laboratory technicians (20.8%). Of all participants, 102 (21.4%) were male and 373 (78.6%) were female.

### Seroprevalence levels

From the sample of HCWs, 73 tested positive for SARS-CoV-2 IgG—a prevalence of 15.37%, and 53 tested positive for SARS-CoV-2 IgM—a prevalence of 11.16%. A total of 189 HCWs (39.79%) reported that they had been previously diagnosed with COVID-19 by a nose swab PCR test. There were overall 231 HCWs who either reported previous PCR diagnosis, or tested positive for serological markers IgG or IgM, which was almost half of the study sample (48.63%, Table 1).

### Effect of other factors on odds of seroprevalence

Analysis of HCWs who were either IgM or IgG positive (Table 2) showed that the odds for being seropositive in SARS-CoV-2 IgG or IgM were increased among male participants in adjusted (OR 2.08, 95% CI 1.20, 3.61) and unadjusted analysis (OR 2.04, 95% CI 1.21, 3.44). HCWs who reported that family members had been diagnosed with COVID-19 also showed higher odds of seropositivity for either antibody in adjusted (OR 2.99, 95% CI 1.78, 5.02) and crude analysis (OR 2.77, 95% CI 1.68, 4.54). Reduced odds of seropositivity were observed for smokers in unadjusted analysis, but adjusted analysis did not reveal any significant association (adjusted OR 0.55, 95% I 0.31, 1.01, crude OR 0.56, 95% CI 0.31, 0.99).

**Table 2:** Odds ratios of IgM or IgG positive HCWs

	Positive		Negative		Crude odds ratio (95% CI)	P value	Adjusted odds ratio (95% CI)	P value
	Events/total (%)		Events/total (%)					
Male	27/83	(32.5)	75/392	(19.1)	2.04 (1.21–3.44)	0.008	2.08 (1.20–3.61)	0.009
Smoker	17/83	(20.5)	124/392	(31.6)	0.56 (0.31–0.99)	0.045	0.55 (0.31–1.01)	0.053
Has infected family members	36/83	(43.4)	85/392	(21.7)	2.77 (1.68–4.54)	< 0.001	2.99 (1.78–5.02)	< 0.001
Member of a family that doesn’t visit restaurants								
False	21/83	(25.3)	73/392	(18.6)	Reference		Reference	
True	62/83	(74.7)	309/392	(78.8)	0.70 (0.40–1.22)	0.205	0.64 (0.35–1.17)	0.146
Don't know/refuse	0/83	(0.0)	10/392	(2.6)	–		–	
Member of family that respects maximally protective measures								
False	3/83	(3.6)	4/392	(1.0)	Reference		Reference	
True	80/83	(96.4)	388/392	(99.0)	0.27 (0.06–1.25)	0.095	0.33 (0.06–1.80)	0.201
Don't know/refuse	0/83	(0.0)	0/392	(0.0)	–		–	

Family behaviour of HCW gave reduced odds of seroprevalence, but the difference was not significant in this case for either being member of families not visiting restaurants (adjusted OR 0.64, 95% CI 0.35, 1.17, crude OR 0.70, 95% CI 0.40, 1.22), or being member of families respecting maximally protective measures (adjusted OR 0.33, 95% CI 0.06, 1.80, crude OR 0.27, 95% CI 0.06, 1.25).

### Effect of other factors on odds of previous PCR diagnosis

In our survey, we examined how odds of reported previous PCR diagnosis were affected by different factors, calculating both crude and adjusted odds ratios (Table 3). Those with larger families, had slightly higher odds of positive PCR test result in crude analysis (OR 1.13, 95% CI 1.01, 1.27), but adjusted analysis did not demonstrate significantly higher odds (OR 1.09. 95% CI 0.96, 1.24). As observed for seropositivity, both crude (OR 2.98, 95% CI 1.95, 4.55) and adjusted (OR 2.82, 95% CI 1.79, 4.44) odds ratios showed that those with infected family members were more likely to have tested positive for COVID-19. Usage of masks by colleagues was not associated with a significant difference in odds of previous diagnosis.

**Table 3:** Odds ratios of PCR positive HCWs

	Positive		Negative		Crude odds ratio (95% CI)	P value	Adjusted odds ratio (95% CI)	P value
	Events/total (%)		Events/total (%)					
Level of education								
High school	59/189	(31.2)	114/286	(39.9)	Reference		Reference	
University degree	78/189	(41.3)	121/286	(42.3)	1.25 (0.82–1.90)	0.310	0.99 (0.61–1.62)	0.971
Postgraduate degree	52/189	(27.5)	51/286	(17.8)	1.97 (1.20–3.24)	0.008	1.24 (0.62–2.48)	0.55
Residence in Prishtina	184/189	(97.4)	249/286	(87.1)	5.47 (2.11–14.2)	< 0.001	4.52 (1.70–12.0)	0.002
Number of family members	4.448421*		1.598711**		1.13 (1.01–1.27)	0.040	1.09 (0.96–1.24)	0.171
Has infected family members	72/189	(38.1)	49/286	(17.1)	2.98 (1.95–4.55)	< 0.001	2.82 (1.79–4.44)	< 0.001
Masks are used by colleagues in the office								
False	2/189	(1.1)	8/286	(2.8)	Reference		Reference	
True	185/189	(97.9)	278/286	(97.2)	2.66 (0.56–12.7)	0.219	3.98 (0.74–21.4)	0.107
Don't know/refuse	2/189	(1.1)	0/286	(0.0)	–		–	
Type of healthcare worker								
Physician	74/189	(39.2)	72/286	(25.2)	Reference		Reference	
Nurse	81/189	(42.9)	149/286	(52.1)	0.53 (0.35–0.81)	0.003	0.52 (0.29–0.93)	0.026
Other	34/189	(18.0)	65/286	(22.7)	0.51 (0.30–0.86)	0.012	0.57 (0.29–1.12)	0.10
Works in the COVID-19 testing unit	95/189	(50.3)	109/286	(38.1)	1.64 (1.13–2.38)	0.009	1.50 (1.00–2.24)	0.05

*Mean

**Standard deviation

Compared with physicians, nurses (adjusted OR 0.52, 95% CI 0.29, 0.93, crude OR 0.53, 95% CI 0.35, 0.81), and other staff (adjusted OR 0.57, 95% CI 0.29, 1.12, crude OR 0.51, 95% CI 0.30, 0.86) had lower odds of seropositivity. Working in the COVID-19 testing unit gave increased odds for a positive PCR test (adjusted OR 1.50, 95% CI 1.00, 2.24, crude OR 1.64, 95% CI 1.13, 2.38).

### Effect of other factors on odds of seropositivity or previous PCR diagnosis

Our examination of COVID-19 overall prevalence, by assessing those who either tested positive for IgG or IgM in the serological tests or had previously been diagnosed with COVID-19 by PCR testing, showed similar patterns to other analyses (Table 4). Males were again found to have significantly higher unadjusted odds of COVID-19 (OR 1.60, 95% CI 1.03, 2.50). Compared with high school graduates, those with higher levels of education had higher odds of COVID-19 prevalence. For HCWs with postgraduate degree, unadjusted analysis gave an odds ratio of 1.96 (95% CI 1.19, 3.21).

**Table 4:** Odds ratios of IgM or IgG or PCR positive HCWs

	Positive		Negative		Crude odds ratio (95% CI)	P value	Adjusted odds ratio (95% CI)	P value
	Events/total (%)		Events/total (%)					
Male	59/231	(25.5)	43/244	(17.6)	1.60 (1.03–2.50)	0.037	1.47 (0.88–2.45)	0.14
Level of education								
High school	72/231	(31.2)	101/244	(41.4)	Reference		Reference	
University degree	99/231	(42.9)	100/244	(41.0)	1.39 (0.92–2.09)	0.117	1.19 (0.74–1.90)	0.470
Postgraduate degree	60/231	(26.0)	43/244	(17.6)	1.96 (1.19–3.21)	0.008	1.38 (0.69–2.73)	0.360
Residence in Prishtina	217/231	(93.9)	216/244	(88.5)	2.01 (1.03–3.92)	0.041	1.59 (0.79–3.21)	0.193
Number of family members	4.448421*		1.598711**		1.14 (1.01–1.28)	0.028	1.08 (0.95–1.22)	0.24
Has infected family members	86/231	(37.2)	35/244	(14.3)	3.54 (2.27–5.53)	< 0.001	3.61 (2.25–5.79)	< 0.001
Masks are used by colleagues in the office								
False	3/231	(1.3)	7/244	(2.9)	Reference		Reference	
True	226/231	(97.8)	237/244	(97.1)	2.23 (0.57–8.71)	0.251	3.38 (0.75–15.1)	0.112
Don't know/refuse	2/231	(0.9)	0/244	(0.0)	–		–	
Type of healthcare worker								
Physician	85/231	(36.8)	61/244	(25.0)	Reference		Reference	
Nurse	99/231	(42.9)	131/244	(53.7)	0.54 (0.36–0.83)	0.004	0.60 (0.34–1.07)	0.081
Other	47/231	(20.3)	52/244	(21.3)	0.65 (0.39–1.08)	0.099	0.71 (0.37–1.38)	0.32
Works in the COVID-19 testing unit	111/231	(48.1)	93/244	(38.1)	1.50 (1.04–2.16)	0.029	1.34 (0.90–1.99)	0.16

*Mean

**Standard deviation

The residence in Prishtina was reflected in higher odds of COVID-19 (adjusted OR 1.59, 95% CI 0.79, 3.21, crude OR 2.01, 95% CI 1.03, 3.92). Those with larger families again showed higher odds of COVID-19 in unadjusted analysis (OR 1.14, 95% CI 1.01, 1.28) which was not significant in adjusted analysis. Infected family members were again associated with significantly higher odds of COVID-19 (adjusted OR 3.61, 95% CI 2.25, 5.79, crude OR 3.54, 95% CI 2.27, 5.53).

Mask usage in co-workers again did not show a significant difference in odds. Those working as nurses or laboratory technicians were again less likely to be infected with COVID-19 compared to physicians, but only the crude odds ratio for nurses (OR 0.54, 95% CI 0.36, 0.83) had had significantly lower odds. Those who worked in COVID-19 testing units also showed higher odds of COVID-19 (adjusted OR 1.50, 95% CI 1.04, 2.16).

## Discussion

Among HCWs, 83 (17.47%) were seropositive for SARS-CoV-2 antibodies IgG or IgM. A total of 189 HCWs (39.79%) reported that they had been previously diagnosed with COVID-19 by a nose swab PCR test. There were overall 231 HCWs who either reported previous PCR diagnosis, or tested positive for serological markers IgG or IgM, which was almost half of the study sample (48.63%, Table 1). Several factors are associated with decreased and increased odds for such outcomes.

Our result of 17.47% seroprevalence is comparable to the range of percentage reported in other similar studies [23–26]. Interestingly, the majority of HCWs who reported having previously tested positive by PCR tests did not test positive for either antibody. Inbaraj et al. reported roughly 7 undetected infections for every PCR confirmed case [27], which is the opposite of the trend we have observed. It is possible that this represents the extensive and thorough testing of HCWs in Prishtina. Alkurt et al. reported that only 78.2% of PCR positive patients had IgG antibodies, and that IgG titres of asymptomatic PCR-positive patients were significantly lower than symptomatic patients. Notably, these patients were tested 52.8 ± 11.6 days after infection. Bendavid’s study in California also showed that the seroprevalence of subjects 2 weeks after symptoms was considerably higher than seroprevalence 2 months after symptoms [28]. It is possible, then, that the low amount of positive antibody tests in comparison with reported PCR tests is due to antibody loss, or to mild or asymptomatic cases among some HCWs. Although it would not account for the full extent of the variation found here, systematic reviews of serological tests have shown considerable variation between accuracy of serological tests, and lower accuracy for serological tests [11, 29, 30].

The study design and the general structure of the study were based on previously published studies [14, 31, 32]. The content of the questionnaire was based on existing studies published and on consultations with healthcare experts, public health experts and staff of the Main Family Medical Centre of Prishtina. The questionnaire was compiled, tested, and revised before data collection. Key healthcare experts and medical staff from the Main Family Medical Centre of Prishtina gave their input on the study design, questionnaire, and overall progress of the study. The main limitation of the study is the relatively small sample size.

Our study is the first study about COVID-19 seroprevalence in HCWs working in Kosovo. Another study by our group has examined the seroprevalence of anti-SARS-CoV-2 antibodies in municipal workers in Prishtina municipality. Out of 418 municpal workers surveyed, we found that 21.1% were seropositive for either IgG or IgM, of which 9.6% were positive for IgM and 19.4% for IgG [33]. There are some studies that previously have examined seroprevalence among HCWs in primary healthcare. In Spain, Barallat et al. found 9.17% of IgG seropositivity in HCWs [22]. Compared to results of other previous studies, in Essen, Germany 1.6% and New York, USA 33% our study stands in the middle of seropositivity among HCWs [34, 35]. HCWs from emergency departments reportedly have a lower percentage of seropositivity (5.9%) [36]. In contrast with our study, Airoldi et al. [37] found that female HCWs have higher odds of seropositivity OR 1.29, 95% CI (0.92 to 1.80), but other studies have shown, like our study, that male HCWs have a higher ORs [26, 38]. Similar to our study, studies have confirmed the increased odds of seropositivity in physicians compared to nurses and other HCWs [38]. Studies have also reported higher odds of seroprevalence for smokers, but without a significant relationship [39].

Our results show that around a fifth of HCWs in Prishtina Municipality have antibodies for COVID-19, and just under half have been infected. This means that there is potential for most HCWs to be infected, which could lead to further spread of COVID and more hospitalization, which again can lead to more risk for HCWs. Considering that newly qualified Kosovar physicians are often going outside Kosovo [40], healthcare staff resources are already limited, and the need for ample health professionals for patients to have sufficient regular care means that protective measures for health professionals are needed. This should alert our policy makers and trigger them to speed up the process vaccinating more people, particularly HCWs, and increase attempts to protect HCWs from burnout.

Our study did not identify a clear link between adherence to protective measures by family, wearing of masks by coworkers, and seroprevalence or odds of diagnosis by PCR. Systematic reviews have suggested that masks have a protective effect on healthcare workers, and for the public in general [41], but higher certainty evidence is still needed [42]. Similarly, the effect of other protective measures has been examined [4, 43], but the effect of family adherence to these measures has not been considered in detail.

In conclusion, we found that several factors increase the odds of IgG and IgM seropositivity. We also found that HCWs who respect basic infection-control measures have lower odds of being seropositive on IgG and IgM, although the difference was not significant. This could be due to collinearity, which was not detected when we ran tests. Further research could clarify the relationship between family adherence to protective measures and usage of masks by staff, and seroprevalence. Our study results could assist policy makers in Kosovo to continue to raise awareness about coronavirus and its health consequences [44]. Further testing in the future would allow observation of how seroprevalence changes over time.

## Data Availability

The datasets used and/or analysed during the current study available from the corresponding author on reasonable request.
